# Bardoxolone Methyl Ameliorates Compression-Induced Oxidative Stress Damage of Nucleus Pulposus Cells and Intervertebral Disc Degeneration *Ex Vivo*

**DOI:** 10.3389/fbioe.2021.814040

**Published:** 2022-02-01

**Authors:** Yueyang Tian, Jiaqi Duan, Yang Cao, Huichao Zhou, Ashish D Diwan, Ji Tu

**Affiliations:** ^1^ School of Medicine, Nankai University, Tianjin, China; ^2^ Queen Mary College, Nanchang University, Nanchang, China; ^3^ Zhengzhou University of Light Industry, Zhengzhou, China; ^4^ Spine Labs, St.George and Sutherland Clinical School, University of New South Wales, Sydney, NSW, Australia

**Keywords:** intervertebral disc degeneration, tissue engineering, compression, oxidative stress, bardoxolone methyl, Nrf2

## Abstract

Intervertebral disc degeneration (IDD) is the main cause of low back pain, and little is known about its molecular and pathological mechanisms. According to reports, excessive compression is a high-risk factor for IDD; compressive stress can induce oxidative stress in nucleus pulposus (NP) cells during IDD progression that, in turn, promotes cell apoptosis and extracellular matrix (ECM) degradation. Currently, NP tissue engineering is considered a potential method for IDD treatment. However, after transplantation, NP cells may experience oxidative stress and induce apoptosis and ECM degradation due to compressive stress. Therefore, the development of strategies to protect NP cells under excessive compressive stress, including pretreatment of NP cells with antioxidants, has important clinical significance. Among the various antioxidants, bardoxolone methyl (BARD) is used to protect NP cells from damage caused by compressive stress. Our results showed that BARD can protect the viability of NP cells under compression. BARD inhibits compression-induced oxidative stress in NP cells by reducing compression-induced overproduction of reactive oxygen species (ROS) and malondialdehyde. Thus, BARD has a protective effect on the compression-induced apoptosis of NP cells. This is also supported by changes in the expression levels of proteins related to the mitochondrial apoptosis pathway. In addition, BARD can inhibit ECM catabolism and promote ECM anabolism in NP cells. Finally, the experimental results of the mechanism show that the activation of the Nrf2 signaling pathway participates in the protection induced by BARD in compressed NP cells. Therefore, to improve the viability and biological functions of NP cells under compression, BARD should be used during transplantation.

## Introduction

The main cause of low back pain (LBP) is intervertebral disc (IVD) degeneration (IDD) (Anderson et al., 2005). LBP is a common, chronic, and expensive musculoskeletal disease that places a huge economic burden on the world (Maniadakis and Gray, 2000). Nucleus pulposus (NP), cartilage endplate, and annulus fibrosus (AF) are interconnected to form IVDs (Fernandez-Moure et al., 2018). The central NP tissue is an important unit for the normal physiological function of the IVD that allows the IVD to maintain a high water content to withstand mechanical stress from all directions (Hutton et al., 1997). There is still no clear explanation for the molecular mechanism of IDD. Many researchers have found that in the harsh environment of IDD, oxidative stress can cause excessive apoptosis of NP cells and disorder of NP extracellular matrix (ECM) metabolism, induce the destruction of normal IVD physiological function and structure, and, finally, lead to the development of IDD (Roughley, 2004; Zhang et al., 2020). Therefore, inhibiting NP cell apoptosis and ECM degradation induced by oxidative stress is of great significance for the treatment of IDD.

At present, the treatment of IDD generally involves discectomy combined with spinal fusion, which can only alleviate the clinical symptoms to a certain extent and cannot completely restore the biological function of IVD. Following the procedure, patients eventually experience recurrence or symptom aggravation (Iatridis et al., 2013). In recent years, NP tissue engineering technology has become a new method for repairing degenerative IVD (Yang and Li, 2009). The goal of NP tissue engineering is to reconstruct the complex structure, including materials and cells, and to replace the degenerative NP tissue. The survival of cells in the harsh environment of IDD is very important (Nomura et al., 2001). In daily life, the spine is subjected to varying degrees of mechanical pressure, and the excessive compression of the IVD, the load-absorbing structure of the spine, is a major cause of IDD (Kang et al., 2020). Therefore, IDD compression models allow investigators to study the pathogenesis of IDD. In the degenerative disc environment, NP cell survival needs to overcome compression-induced injury (Wang et al., 2020c). A previous study has shown that when the pressure reaches 1.0 MPa, mitochondrial dysfunction, excessive ROS production, and apoptosis are observed NP cells (Hutton et al., 1999).

Bardoxolone methyl (BARD) is a synthetic triterpenoid. In studies of diabetic nephropathy and acute lung injury, it has been confirmed that BARD can exert an antioxidant effect by activating the Nrf2/ARE pathway (Nagasu et al., 2019; Pei et al., 2019; Rossing et al., 2019; Kanda and Yamawaki, 2020). However, it is unclear whether BARD can inhibit compression-induced oxidative stress, apoptosis, and ECM degradation in NP cells. Therefore, we investigated the effect of BARD on NP cell injury induced by compression and the underlying molecular mechanism. This experiment is of great significance for optimizing the application of NP tissue engineering in the treatment of IDD.

## Materials and Methods

### Human NP Cell Acquisition and Culture

Human NP cells, derived from human IVD NP, were purchased from ScienCell Research Laboratory (ScienCell). NP cells were cultured as described below (Kang et al., 2017). Briefly, NP cells were maintained in a mixed medium containing DMEM/F12 (Gibco), supplemented with 15% fetal bovine serum (FBS) and 1% penicillin/streptomycin (P/S) (Invitrogen), and placed in a 5% CO_2_ incubator at 37°C. In order to keep the phenotype stable, we used second-generation cells for the experiments.

### Cell Viability Assay

According to the manufacturer’s instructions, a cell counting kit (CCK-8; Biosharp) was used for cell viability analysis. Briefly, after treatment with different concentrations of BARD (MCE; 99.72%) treatment or compression treatment, the cells were seeded in a 96-well plate and subjected to different conditions. Then, 10 μl of CCK-8 solution was added, and the samples were incubated at 37°C for 2 h. Finally, a spectrophotometer was used to measure absorbance at 450 nm.

### Compression Treatment

The tissue or cells were placed, in cell culture plates, at the bottom of the compression device, which was then placed in a 37°C incubator. The compression device is pressurized until the pressure reaches 1.0 MPa (Kang et al., 2020). The control samples were not placed in the compression device during culture. The specific operation was performed as described previously (Iatridis et al., 2013).

### Western Blotting

According to the manufacturer’s instructions, a nuclear and cytoplasmic protein extraction kit (Beyotime) was used to extract total protein, cytoplasmic protein, and nuclear protein from NP cells and tissues. The BCA protein analysis kit (Beyotime) was used to determine the protein concentration. Equal amounts of protein from each sample were separated using SDS-PAGE and transferred to a PVDF membrane. Non-fat milk (5%) was used to block the membranes at room temperature for 2 h. Then, the membranes were incubated overnight at 4°C with the following primary antibodies: Bax (Abcam), Bcl-2 (Abcam), cleaved caspase-9 (Abcam), cleaved caspase-3 (Cell Signaling Technology), cytochrome c (Abcam), collagen II (Abcam), MMP-13 (Thermo Fisher), Nrf2 (Abcam), and HO-1 (Proteintech). Histone (Abcam) and GAPDH (Cell Signaling Technology) were used as the internal controls. Subsequently, the membrane was washed with TBST and incubated with the respective secondary antibodies (Abcam) for 1 h at room temperature, and then, the membranes were washed with TBST again. Protein bands were observed by enhanced chemiluminescence (Thermo Fisher) according to the manufacturer’s instructions. ImageJ software (NIH) was used to quantify band intensity.

### Flow Cytometry

The cells were digested with trypsin (Solarbio) without EDTA, washed twice with PBS, and stained with Annexin V–FITC and PI for 20 min (Keygen, China). Then, they were immediately analyzed using a FACSCalibur flow cytometer (BD Biosciences).

### Measurements of ROS and Malondialdehyde Levels

The cells were processed according to the experimental design using ROS (Beyotime) and MDA (Beyotime) kits to determine the content of ROS and MDA, respectively, in human NPCs according to the manufacturer’s instructions.

### Immunofluorescence

The immunofluorescence assay was performed following different cells, according to a previously described procedure. The assay was performed through incubating the samples with antibodies against MMP-13 (Thermo Fisher) overnight at 4°C, followed by incubation with Alexa Fluor^®^ 488-conjugated secondary antibodies for 1 h at 37°C. The nuclei were stained with 4′,6-diamidino-2-phenylindole (Beyotime). Finally, each slide was observed under a fluorescence microscope, and the fluorescence intensity was quantified using ImageJ software.

### siRNA Transfection

Small interfering RNA (siRNA) that was against Nrf2 (si-Nrf2) mRNA was produced by Gene Pharma. According to the manufacturer’s instructions, Lipofectamine^®^ 2000 (Invitrogen) was used to transfect NP cells with 100 nmol/L of each siRNA for 6 h. After 48 h, the cells were used for the experiments.

### *Ex Vivo* IVD Organ Culture

12-week-old Sprague–Dawley (SD) rats were used for the IVD. The tail disc was separated and cultivated with a complete endplate structure in DMEM containing 15% FBS (Gibco) and 1% P/S (Invitrogen). The specific operation was as described previously (Wu et al., 2019).

### Assessments of the *Ex Vivo* IVD Compression Model

After treatment, the IVD tissues of SD rats were fixed in formaldehyde, decalcified, dehydrated, embedded in paraffin, and cut into slices, with a thickness of 4 μm. These sections were stained with hematoxylin and eosin (HE) and safranin O-fast green (SO). According to previously described methods, the histological score was used to assess the degree of IVD injury (Han et al., 2008). For immunohistochemical analysis, sections were incubated with Nrf2 primary antibodies at 4°C overnight and then incubated with appropriate horseradish peroxidase-conjugated secondary antibodies and counterstained with hematoxylin. Images were captured using an optical microscope (Olympus).

### Statistical Analyses

The results are expressed as mean ± standard deviation (SD). At least three independent experiments were performed for each test. SPSS software (version 20.0; IBM Corporation) was used to analyze the data. Student’s t-test or analysis of variance was used, followed by Tukey’s test, to assess the differences between the results of each group. Statistical significance was set at *p* < 0.05.

## Results

### The Protective Effect of BARD on the Viability of NP Cells Induced by Compression

**FIGURE 1:**
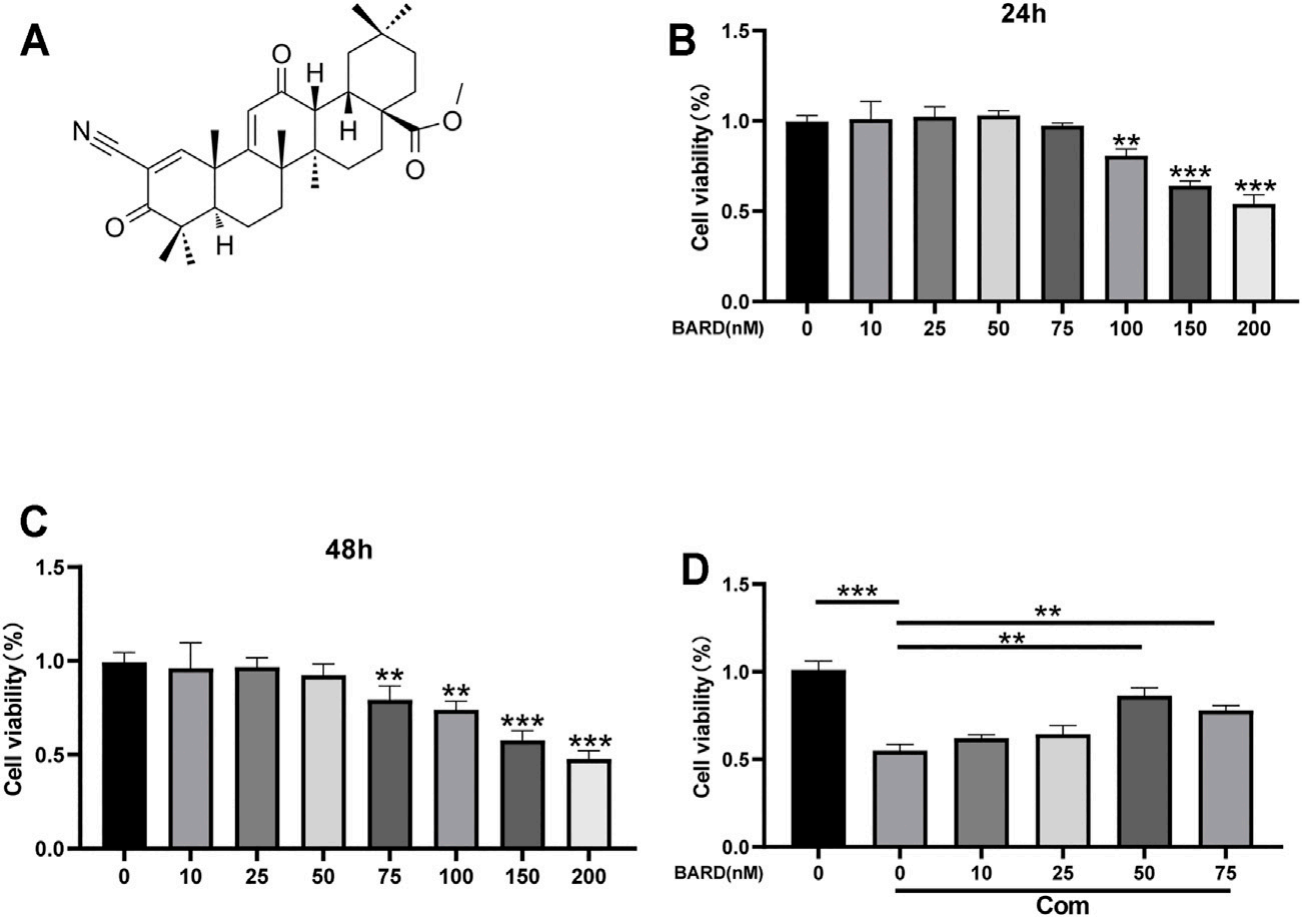
The effect of BARD on NP cell viability. **(A)** Molecular structure of BARD. CCK-8 assay is used to detect changes in the viability of NP cells treated with different concentrations of BARD for 24 h **(B)** and 48 h **(C)**. **(D)** 50 and 75 nM BARD significantly improve NP cell viability under compression. Data are expressed as mean ± SD. ***p < 0.001, **p < 0.01, n = 3.

Compression (Com) was used to establish an IDD model *in vitro*. Figure 1A shows the BARD structural formula. The results of the CCK-8 assay showed that at ≤50 nM, BARD was not cytotoxic to human NP cells for 24 and 48 h treatment times (Figures 1B,C). Subsequently, we observed that under compression conditions, 50 nM BARD showed the best cytoprotective effect (Figure 1D). Therefore, BARD was used at a dose of 50 nM in subsequent experiments.

### The Protective Effect of BARD on Compression-Induced Oxidative Stress in NP Cells

**FIGURE 2:**
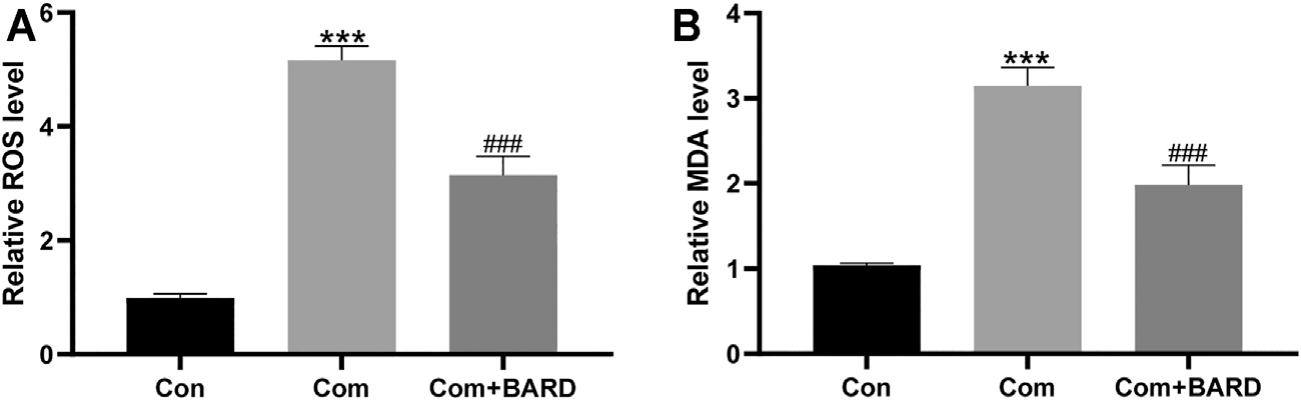
The effect of BARD ROS and MDA accumulation in NP cells caused by compression. **(A–B)** The ROS and MDA contents of NP cells were detected with a fluorescence microplate reader. Data are expressed as mean ± SD. *** indicates *p* < 0.001 when data are compared to those for the control (Con) group. ^###^ indicates *p* < 0.001 when data are compared to those for the compression (Com) group. n = 3.

To assess the level of oxidative damage, we tested the levels of ROS and MDA, which are commonly used indicators of oxidative stress. Compression increased the levels of ROS and MDA in NP cells compared to the control (Con) group (Figures 2A,B). Compared with compression treatment alone, BARD treatment significantly reduced ROS and MDA levels, which suggests that BARD protects NP cells from oxidative stress.

### The Protective Effect of BARD on Compression-Induced Apoptosis in NP Cells

**FIGURE 3:**
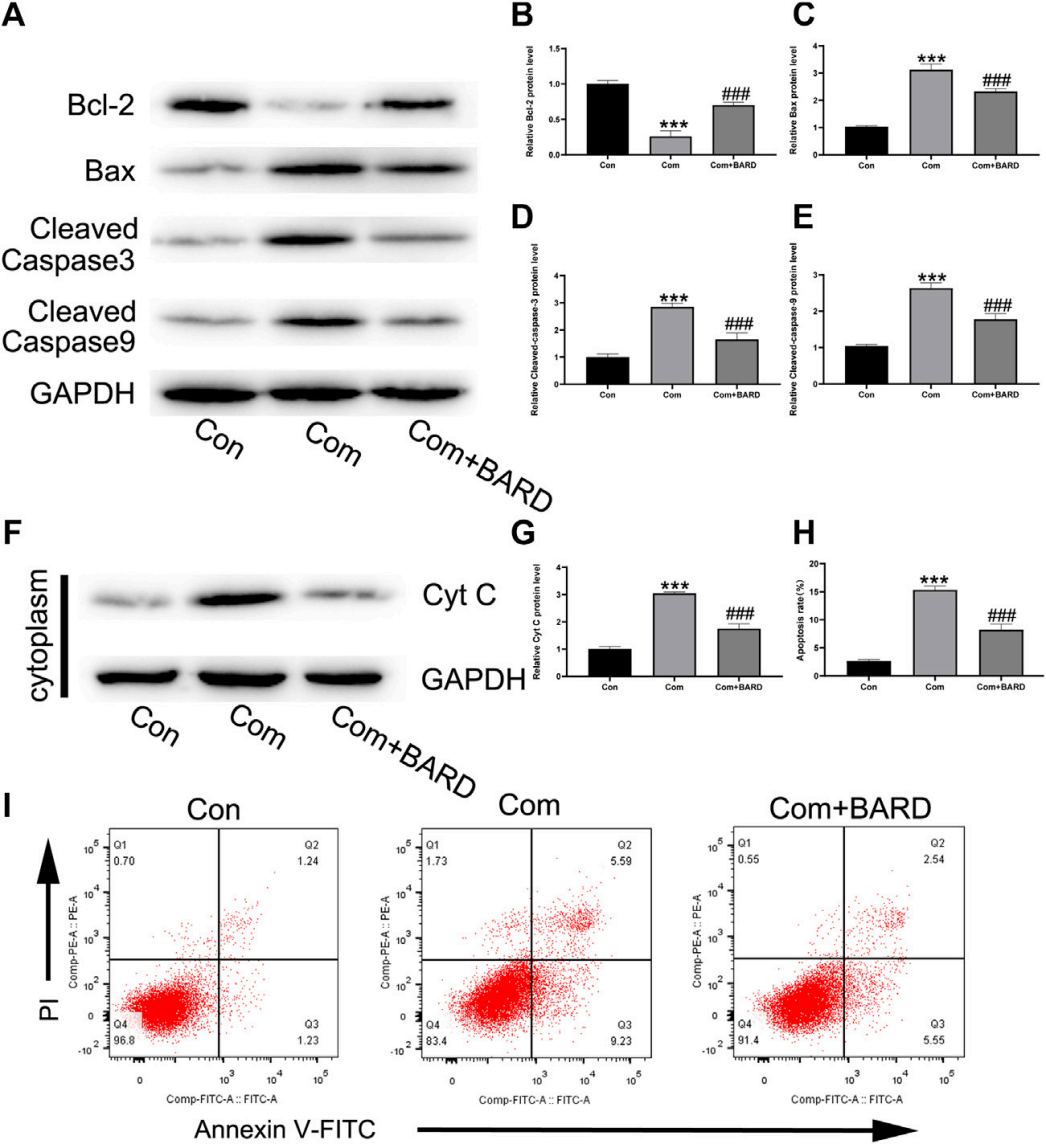
BARD prevents nucleus pulposus cell apoptosis caused by compression. **(A–E)** The expression of BCL2, BAX, cleaved caspase-3, and cleaved caspase-9 in NP cells after BARD treatment was detected by Western blotting. **(F–G)** The expression of cytochrome c (cyt c) in the cytoplasm decreased after BARD treatment, indicating that BARD relieves NP cell apoptosis through the mitochondrial apoptotic pathway. **(H–I)** Flow cytometry results show the apoptosis rate of NP cells. Data are expressed as mean ± SD. *** indicates *p* < 0.001 when data are compared to those for the control (Con) group. ^###^ indicates *p* < 0.001 when data are compared to those for the compression (Com) group. n = 3.

Western blotting was used to detect changes in apoptosis-related proteins in the mitochondrial pathway after BARD treatment (Figure 3A). The results showed that after BARD treatment, the expression of the anti-apoptotic protein Bcl-2 increased, while that of the pro-apoptotic protein Bax decreased (Figures 3B,C). In addition, the expression of cleaved caspase-3 and cleaved caspase-9 in the BARD treatment group was lower than that in the compression group (Figures 3D,E). In addition, the level of cytochrome c in the cytoplasm changed significantly due to BARD treatment (Figures 3F,G). As shown in Figures 3H,I, we used Annexin V–FITC/PI staining to detect the apoptosis of NP cells. The flow cytometry results showed that compression significantly increased the number of apoptotic NP cells. However, BARD treatment significantly alleviated this trend.

### The Protective Effect of BARD on Compression-Induced ECM Degradation in NP Cells

**FIGURE 4:**
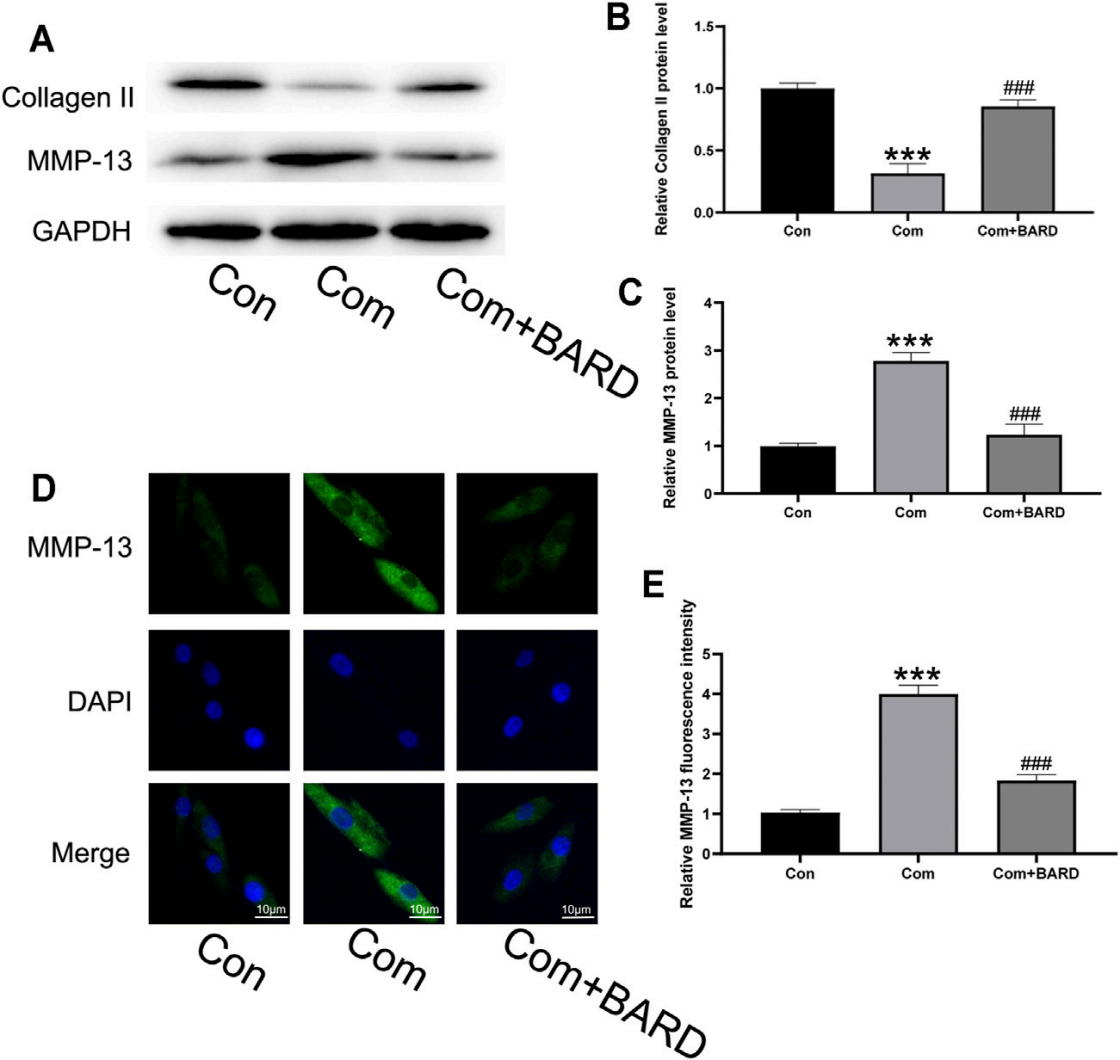
BARD prevents nucleus pulposus cell ECM degeneration caused by compression. **(A–C)** Western blotting was used to detect collagen II and MMP-13 expression. **(D–E)** Immunofluorescence was used to observe the expression of MMP-13. Data are expressed as mean ± SD. *** indicates *p* < 0.001 when data are compared to those for the control (Con) group. ^###^ indicates *p* < 0.001 when data are compared to those for the compression (Com) group. n = 3.

Because the imbalance between ECM synthesis and degradation is also an important feature of IDD, we evaluated collagen II (main ECM component) and MMP-13 (ECM catabolism factor) protein expression levels. As shown in Figures 4A–C, compression treatment decreased collagen II and increased MMP-13 protein levels, and these compression-induced alterations were significantly ameliorated by BARD pretreatment. The immunofluorescence staining results showed that MMP-13 levels significantly increased under compression and BARD ameliorated this trend (Figures 4D,E). These results suggest that BARD protects against compression-induced ECM degeneration.

### The Protective Effect of BARD on the Nrf2 Pathway in Compression-Treated NP Cells

**FIGURE 5:**
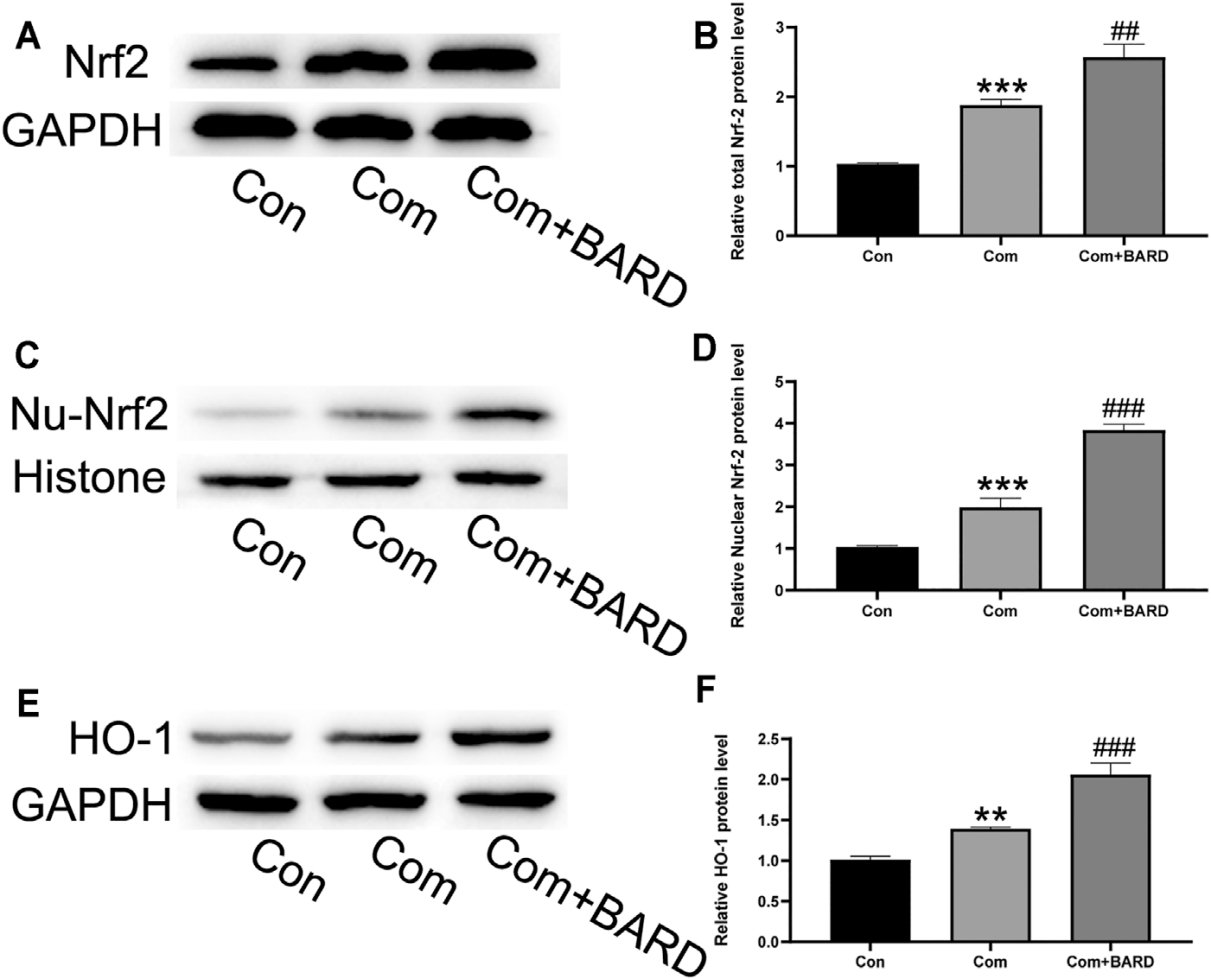
The effect of BARD on the Nrf2 pathway in compression-exposed NP cells. **(A–B)** Western blotting was used to detect Nrf2 expression. **(C–D)** Western blotting was used to detect nuclear Nrf2 (Nu-Nrf2) expression. **(E–F)** Western blotting was used to detect HO-1 expression. Data are expressed as mean ± SD. *** indicates *p* < 0.001 and ** indicates *p* < 0.01 when data are compared to those for the control (Con) group. ^###^ indicates *p* < 0.001 and ^##^ indicates *p* < 0.01 when data are compared to those for the compression (Com) group. n = 3.

As shown in the above results, oxidative stress, apoptosis, and ECM degeneration in NP cells caused by compression were all significantly alleviated after BARD treatment. BARD has been found to activate the Nrf2 pathway in many studies, and as a classic antioxidative stress-related pathway, the Nrf2 pathway plays an important role in compression-induced NP cell damage. Therefore, it is reasonable to assume that the Nrf2 pathway is involved in the protection of BARD against compression-induced NP cell damage. To verify our hypothesis, we evaluated Nrf2 signaling pathway activation under different processing conditions. Compression increased Nrf2 expression (Figures 5A,B) and nuclear translocation of Nrf2 (Figures 5C,D), and the expression of its downstream target protein HO-1 also increased (Figures 5E,F). BARD treatment further increased Nrf2 pathway activation in NP cells induced by compression. Thus, the Nrf2 pathway is implicated in the protective effect of BARD on NP cells.

### Nrf2 Activation is Required for BARD-Induced NP Cell Protection Against Compression

**FIGURE 6:**
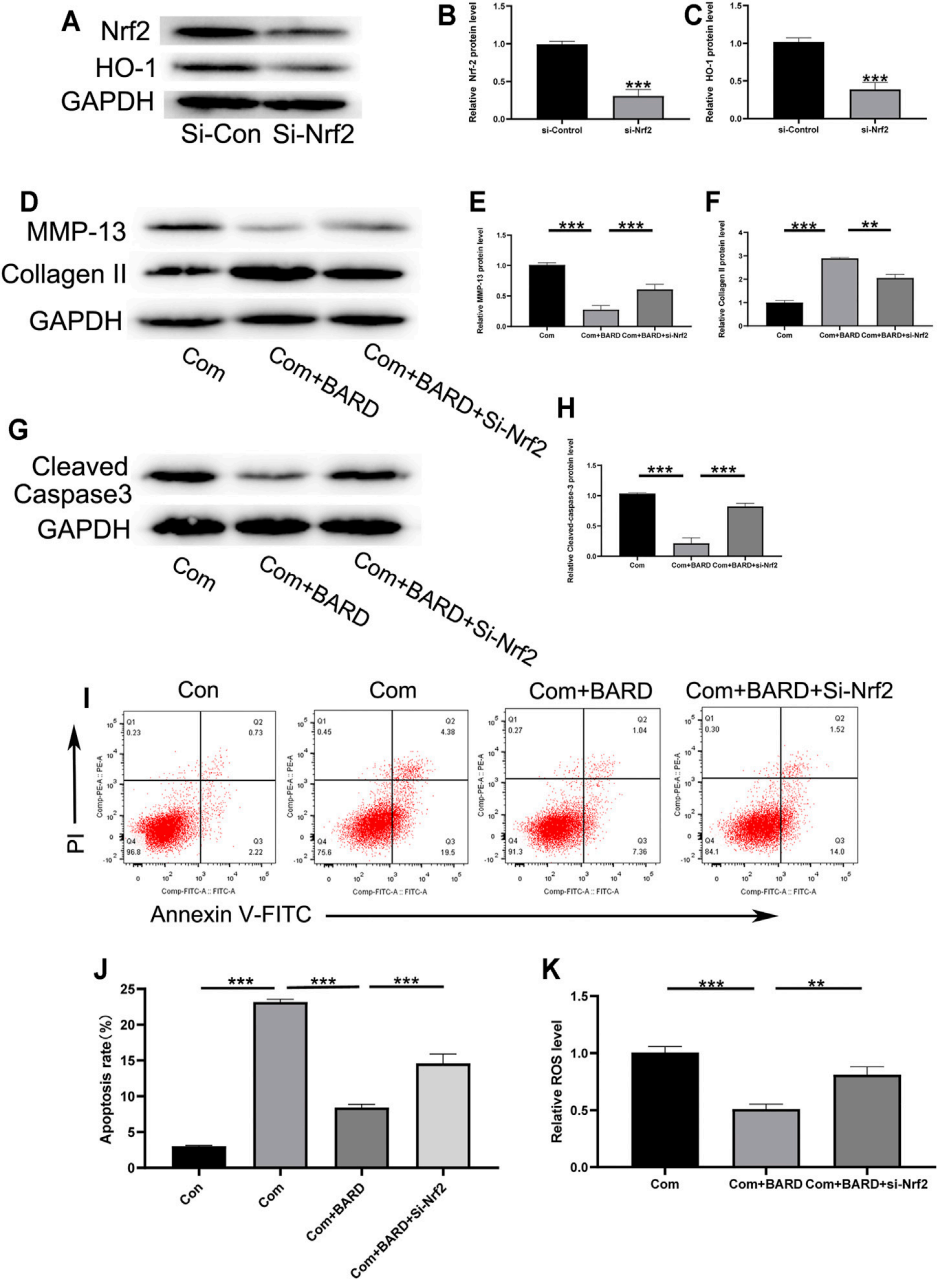
Nrf2 signaling plays a role in the BARD antioxidative stress response in compressed-exposed NP cells. **(A–C)** Western blotting was used to detect the expression of Nrf2 and HO-1. **(D–F)** Western blotting was used to detect the expression of MMP-13 and collagen II. **(G–H)** Western blotting was used to detect the expression of cleaved caspase-3. **(I–J)** Flow cytometry results show the apoptosis rate of NP cells. **(K)** The ROS content of NP cells was detected with a fluorescence microplate reader. ***p < 0.001, **p < 0.01. n = 3.

The present results show that BARD activates the Nrf2 cascade and protects human NP cells from compression-induced cell injury. We further studied the link between Nrf2 activation and BARD-induced cytoprotection in NP cells. As shown in Figures 6A,B, si-Nrf2 significantly knocked down the expression level of Nrf2 in NP cells. Furthermore, the expression of Nrf2-dependent genes (HO-1) was significantly blocked by Nrf2 knockdown (Figure 6C). Importantly, the protection of BARD against compression-induced oxidative injury in human NP cells was reversed through Nrf2 knockdown, as indicated by collagen II, MMP-13, cleaved caspase-3, apoptosis, and ROS levels (Figures 6D–K). These results suggest that Nrf2 activation is required for BARD-induced cytoprotection in compression-treated NP cells.

### BARD Ameliorates NP Tissue Degeneration in an *Ex Vivo* IDD Model

**FIGURE 7:**
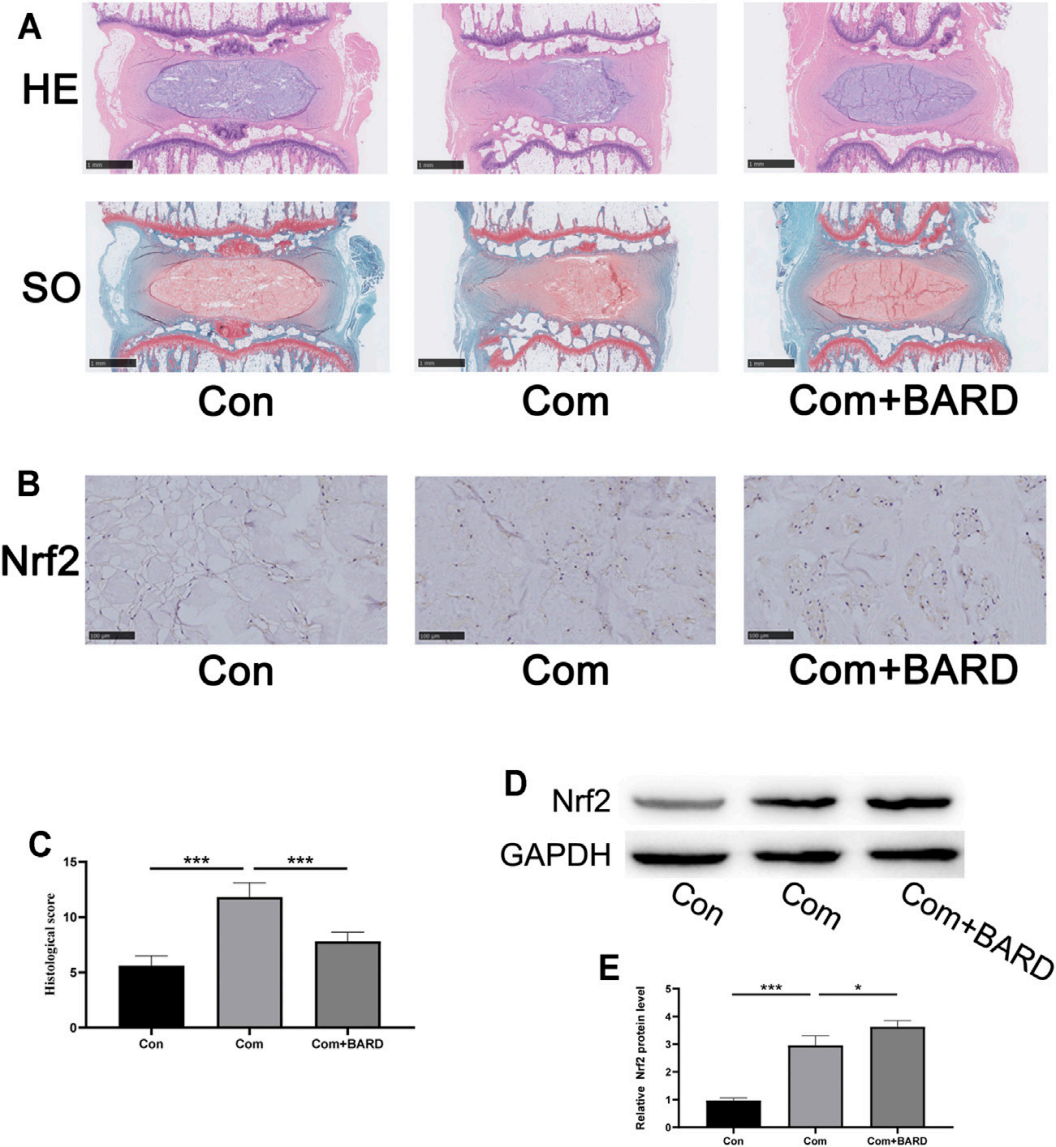
BARD improves the development of IDD in an *ex vitro* compression model. **(A)** HE and SO staining of the rat IVD tissues. Scale bar: 1 mm. **(B)** Immunohistochemical staining showing Nrf2 protein expression in NP tissues. Scale bar: 100 μm. **(C)** The histological score of SD rat IVD tissue was based on histological grading scale. **(D–E)** Western blotting was used to detect Nrf2 expression. Data are expressed as mean ± SD. *** indicates *p* < 0.001 when data are compared to those for the control (Con) group. ^###^ indicates *p* < 0.001 when data are compared to those for the compression (Com) group. n = 5.

We used the compressed isolated rat tail disc degeneration model to further confirm the results of the above *in vitro* experiments. After 2 weeks of compression treatment, the collected disc tissues were stained with HE and SO to evaluate morphological changes (Figure 7A). The compression treatment group showed severe degenerative changes, but the BARD treatment group significantly alleviated this process. The histological score further proved that BARD could prevent the IDD process (Figure 7C). Based on the results of the *in vitro* studies, *ex vivo* Nrf2 activation by BARD was further verified. Consistent with the results of the *in vitro* studies, the immunohistological staining (Figure 7B) and Western blotting (Figures 7D,E) results showed that BARD promoted Nrf2 expression in NP tissues. These results indicate that BARD ameliorated rat tail disc degeneration caused by compression. At the same time, the protective effect of BARD may be mediated by Nrf2 upregulation.

## Discussion

Previous studies have shown that excessive compression plays an important role in the development of IDD (Cheng et al., 2021; Lyu et al., 2021). Compression induces oxidative stress, apoptosis, and ECM degradation in NP cells (Li et al., 2018). Oxidative damage can promote apoptosis and ECM degradation, which contribute to the development of IDD (Chen et al., 2019; Xu et al., 2021). Therefore, for the successful application of NP tissue engineering, we focused on preventing compression-induced oxidative stress and subsequent NP cell apoptosis and ECM degradation (Sun et al., 2015). This may be a valuable method for alleviating and reversing IDD progression. Our study showed that BARD effectively increased the viability of NP cells treated with compression. In terms of indicators of oxidative stress, BARD prevented the production of excessive ROS and MDA in NP cells induced by compression. This study also showed that BARD could prevent compression-induced mitochondrial apoptosis in NP cells. In terms of ECM metabolism, BARD can inhibit ECM degradation and promote ECM synthesis. In terms of molecular mechanisms, BARD can promote the nuclear transfer of Nrf2 proteins and the overexpression of Nrf2 target proteins, which may reflect the molecular basis of the antioxidant effect of BARD. The *ex vivo* compression model also showed that BARD could reduce progressive damage of the IVD structure induced by compression.

Intracellular oxidative stress is precisely regulated and slightly biased toward oxidative processes (Balaban et al., 2005). Due to the transfer of electrons during oxidative phosphorylation, ROS are inevitably produced. As a by-product of aerobic catalysis, ROS levels are often used as an indicator of oxidative stress (Giorgio et al., 2005). The main forms of ROS include hydrogen peroxide (H_2_O_2_), superoxide anions (O_2_^−^), and free radicals. Lower concentrations of ROS act as signaling molecules to activate specific physiological pathways that control multiple life processes (Finkel and Holbrook, 2000; Quarrie and Riabowol, 2004). When the balance between the production and removal of ROS in the body is disrupted, the increase in ROS levels destroys DNA, proteins, and lipids, eventually triggering oxidative stress and leading to cell damage (Glasauer and Chandel, 2013; Tsukahara, 2007). Recent studies have shown that the occurrence and development of IDD is closely related to ROS and oxidative stress (Dimozi et al., 2015; Hou et al., 2014; Suzuki et al., 2015). Oxidative stress can accelerate the process of IDD in many ways, including through apoptosis, ECM degeneration, senescence, and autophagy (Feng et al., 2017). Many reports have shown that BARD has a strong antioxidative effect (Khurana et al., 2020; Pang et al., 2021; Snijders et al., 2021). However, whether BARD can alleviate IVD degeneration caused by compression has not been studied. This experiment proved that BARD can significantly reduce the increase in ROS and MDA levels caused by compression.

Under normal conditions, apoptosis plays an important role in maintaining tissue homeostasis. Apoptosis is a self-programmed cell destruction process. Its purpose is to remove unwanted cells and remodel development (Zhang et al., 2021). A major cause of IDD is the excessive apoptosis of IVD cells (Ding et al., 2013; Zhang et al., 2021). Excessive ROS increase the permeability of the outer mitochondrial membrane and the release of the pro-apoptotic factor, cytochrome c, which leads to cell apoptosis (Pervaiz et al., 2009). The important physiological function of IVD cells is to secrete ECM components (Tsingas et al., 2020). The ECM surrounds the IVD cells, maintains their normal physiological functions and characteristics, and, ultimately, maintains the normal physiological structure and tissue stability of the IVD. The ECM also provides buffering capacity to resist compressive mechanical loads on the spine from all directions (Li et al., 2021). However, during the IDD process, many factors, such as aging, inflammation, oxidative stress, and abnormal pressure load, lead to imbalances in the synthesis and degradation of the ECM components in the IVD tissue, and eventually, the IVD irreversibly degenerates (Wang et al., 2020b). In particular, the NP tissue is located at the center of the IVD, and compression leads to the destruction of the IVD tissue, which causes the ability of the IVD to withstand mechanical loads to weaken. Furthermore, the physiological stress structure of the spine changes, leading to a series of spinal degenerative diseases (Wang et al., 2020b; Kim et al., 2021; Zhang et al., 2021). Therefore, preventing NP cell apoptosis and ECM degradation induced by oxidative stress may be effective methods for the treatment of IDD.

Nrf2 is a transcription factor that plays an important role in the cell response to oxidative stress. Nrf2 activation represents the initiation of the oxidative stress defense system. In the non-stimulated state, Nrf2 exists in the cytoplasm, and the Nrf2 protein contains two Keap1 protein-binding motifs (Tu et al., 2019). ETGE and DLG enable Nrf2 to bind to the inhibitory protein Keap1 in the cytoplasm. Keap1 functions as an adaptor for cullin 3 (CUL3) E3 ubiquitination ligase-mediated Nrf2 proteasomal degradation. The cysteine residues on Keap1 can be modified by oxidants or electrophiles, which cause the protein to undergo conformational changes, leading to the dissociation of Keap1–Nrf2, the termination of Nrf2 polyubiquitination, and the translocation of Nrf2 into the nucleus (Ferrándiz et al., 2018). Nrf2 combines with the sMaf (small musculoaponeurotic fibrosarcoma) protein to form a heterodimer, which can then combine with antioxidant response elements to initiate the transcription of multiple target genes that are involved in redox balance, metabolic response, inflammatory response, etc. (Alcaraz and Ferrándiz, 2020). The antioxidative stress effect of Nrf2 has been reported in many studies of IDD, and Nrf2 in NP cells can directly act through its downstream target regulated antioxidant proteins (Kang et al., 2020; Wang Y. et al., 2019). On the other hand, the mitochondrion is the body’s energy factory, and it is also the main source of ROS (Fukai and Ushio-Fukai, 2020; Zhang and Wong, 2021). Nrf2 can reduce the production of ROS from the source by maintaining mitochondrial homeostasis, thereby maintaining the redox balance in NP cells (Tang et al., 2019; Wang K. et al., 2019). Various drugs can inhibit IDD by activating the Nrf2 signaling pathway (Gu et al., 2019; Song et al., 2021; Wang K. et al., 2019; Wang et al., 2020a; Zhong et al., 2021). In this study, we confirmed that BARD increased the expression and nuclear translocation of Nrf2 and downstream HO-1 expression also significantly increased. In addition, inhibition of Nrf2 attenuated the protective effect of BARD on oxidative stress damage in NP cells.

There were several limitations associated with our study. It is well known that NP cells are in a hypoxic state *in vivo*. However, the oxygen concentration of cells cultured *in vitro* in this experiment was normal, which caused excessive ROS production. Second, this experiment only explored the effect of BARD on Nrf2 activation. Whether BARD affects the activation of other pathways has yet to be determined.

In conclusion, this study provides evidence that BARD protects NP cells from apoptosis and ECM degradation under compression-induced oxidative stress. Its protective effect is, at least partially, mediated by the Nrf2 signaling pathway. Therefore, to improve the effectiveness of NP tissue engineering, BARD has been proposed as a supplement for minimizing the destructive effect of compression on engineered NP tissue.

## Data Availability

The raw data supporting the conclusions of this article will be made available by the authors, without undue reservation.
